# Experimental investigation of sludge dewatering for single- and double-drainage conditions with a vacuum negative pressure load at the bottom

**DOI:** 10.1371/journal.pone.0253806

**Published:** 2021-06-28

**Authors:** Jiesheng Zhang, Yongzheng Qi, Xue Zhang, Guofu Zhang, Hang Yang, Firdawus Nattabi

**Affiliations:** 1 The First Engineering Co., LTD. of CTCE Group, Hefei, China; 2 School of Civil Engineering and Architecture, Jiangsu University of Science and Technology, Zhenjiang, China; 3 School of Traffic & Transportation Engineering, Changsha University of Science and Technology, Changsha, China; China University of Mining and Technology, CHINA

## Abstract

The moisture content of municipal sludge is relatively high, which increases the cost of sludge transportation and treatment. To reduce the volume of the sludge, sludge dewatering is needed. This paper proposes the theory of sludge dewatering and facilitates efficient and economical technology of sludge dewatering. Sludge dewatering tests were carried out by using homemade rapid sludge dewatering devices. There were two groups of tests with single- and double-drainage conditions, and all test runs were loaded with a negative vacuum pressure at the bottom. During the experiments, the vacuum degree and the pore water pressure in the sludge were monitored in real time. After the experiments, the data were compared and analyzed. At the initial stage, the sludge dewatering extent and the sludge dewatering velocity for double-drainage conditions were much higher than those for single-drainage conditions. The vacuum occurring for single-drainage conditions lagged behind that for double-drainage conditions in the sludge. The value of vacuum degree for single-drainage conditions was lower than that for double-drainage conditions, and the vacuum attenuation for single-drainage conditions was considerable. The excess pore water pressure for double-drainage conditions dissipated faster than that for single-drainage conditions in the sludge. The pore water pressure for single-drainage conditions at the top and middle of the sludge layer first increased and then decreased in the early loading stage, resembling the Mandel effect. Overall, with a vacuum negative pressure load at the bottom, the sludge dewatering efficiency for double-drainage conditions was much higher than that for single-drainage conditions. This study provides an experimental and theoretical basis for engineering applications in the sludge treatment industry.

## Introduction

The moisture content of municipal sludge is very high. The sludge moisture content in the primary sedimentation tank is generally between 95% and 97%, and the moisture content of the excess sludge in the secondary sedimentation tank is more than 99%. Therefore, it is necessary to implement techniques to reduce sludge volume, such as concentration, dehydration, and drying [1]. The primary treatment method for sludge volume reduction is sludge dewatering, so the effect of sludge dewatering has a direct impact on the degree of sludge reduction [2]. Reducing the sludge moisture content is the key to the subsequent treatment and disposal of sludge [3,4]. The sludge volume can be significantly reduced through sludge dewatering treatment. Thus, the cost of sludge transportation and treatment will be reduced [5]. However, sludge has a complex composition, strong hydrophilicity, and a high water content, which makes sludge dewatering difficult [6–8]. Therefore, rapid sludge dewatering is a challenge in sludge treatment [9]. Sludge dewatering methods are mainly carried out by mechanical means such as belt type, plate frame type, and centrifugal type [10]. After dewatering, the sludge is gelatinous, and the moisture content is approximately 80% [11].

**Fig 1:**
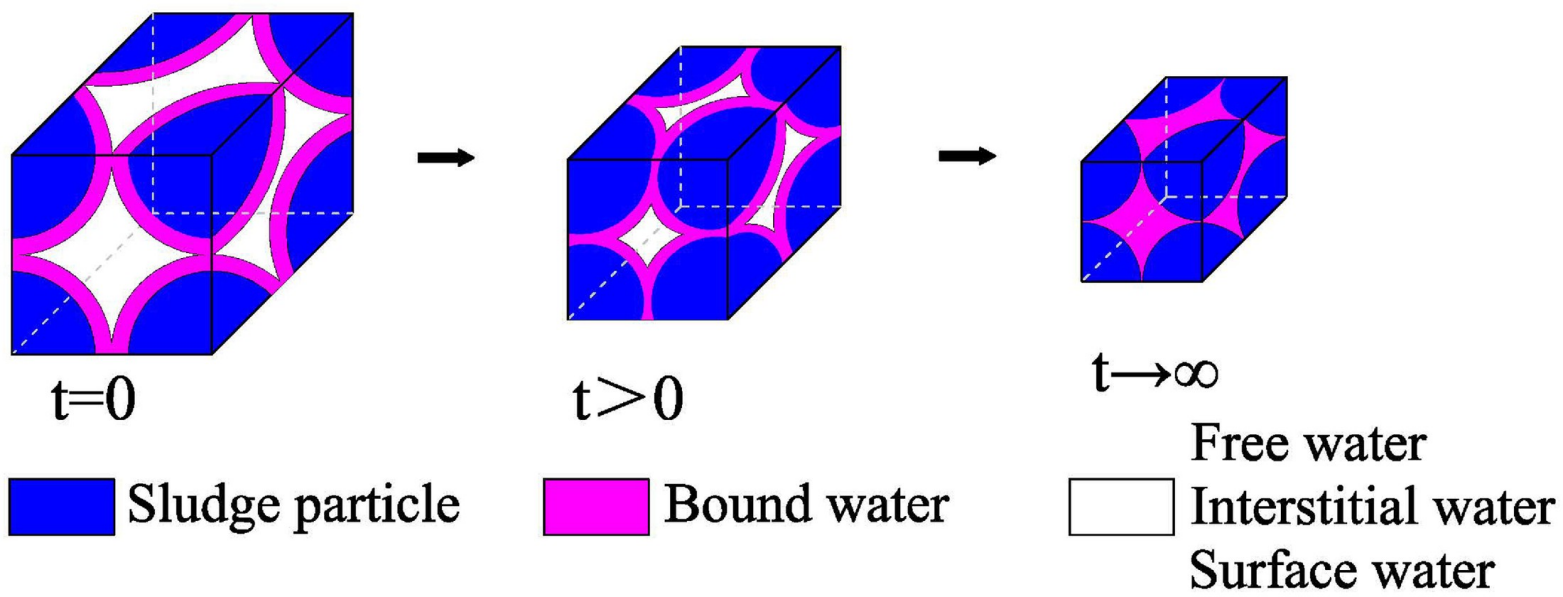
The process of sludge dewatering.

Sludge dewatering is a process in which the sludge’s internal water is discharged from the drainage channel driven by an external force, and the sludge dewatering process is shown in Fig 1 [12]. Due to the solid sludge particle characteristics, such as high hydrophilicity, difficult sedimentation, and strong compressibility, the efficiency and effect of sludge dewatering are related to the sludge dewatering treatment process. Different dewatering treatment processes show different treatment efficiencies and effects. Therefore, it is particularly important to study sludge dewatering theory and develop an efficient and economic technology for sludge dewatering reduction [13–15]. The bottom vacuum sludge dewatering process is simple and inexpensive. In this study, a comparative experimental study on sludge dewatering for single- and double-drainage conditions with a vacuum negative pressure load at the bottom was carried out.

## 1. Materials and methodology

### 1.1. Materials

The sludge with an initial moisture content of 97.5% used in the test was from the excess sludge of the Zhenjiang sewage treatment plant in Jiangsu Province. The geotextile was a nonwoven permeable geotextile. The parameters of the permeable geotextile used in the test are shown in Table 1.

**Table 1:** Parameters of the permeable geotextile.

Aperture (mm)	thickness (mm)	Surface density (g/m^2^)	permeability coefficient *k*_*v*_ (cm/s)
0.071	5.21	613	0.312

**Fig 2:**
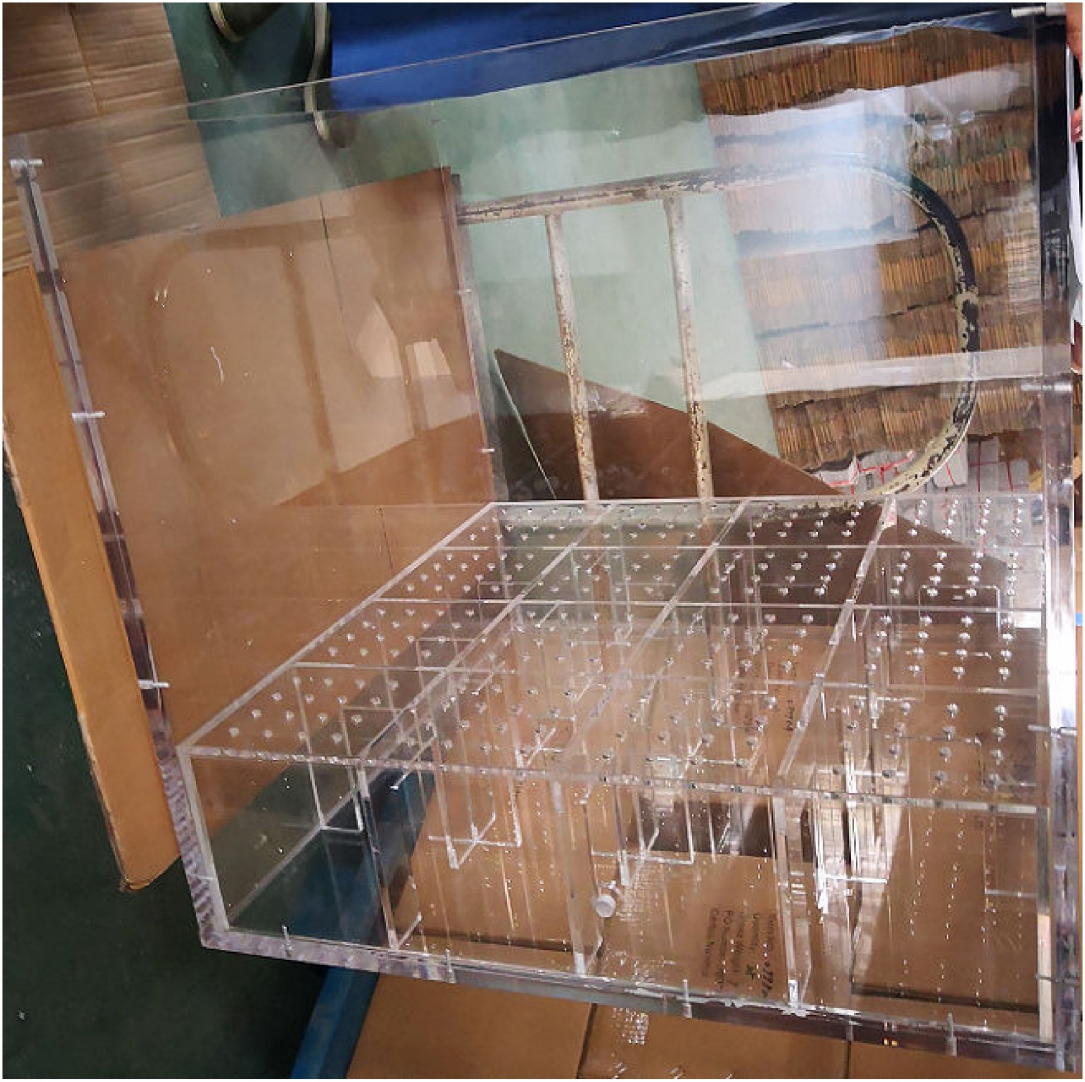
Acrylic model box.

The model box used in the test was made of transparent acrylic material. The model box’s inner net size was 80 cm × 80 cm × 80 cm, and the box wall thickness was 2 cm. The bottom of the model box was set as a hollow space, on which there was a transparent acrylic partition. The partition board had holes with a spacing of 4 cm and a diameter of 1 cm. As shown in Fig 2.

### 1.2. Methodology

The tests were divided into two groups: sludge dewatering for single-drainage conditions and double-drainage conditions with a vacuum negative pressure load at the bottom.

#### 1.2.1. Sludge dewatering for single-drainage conditions

**Fig 3:**
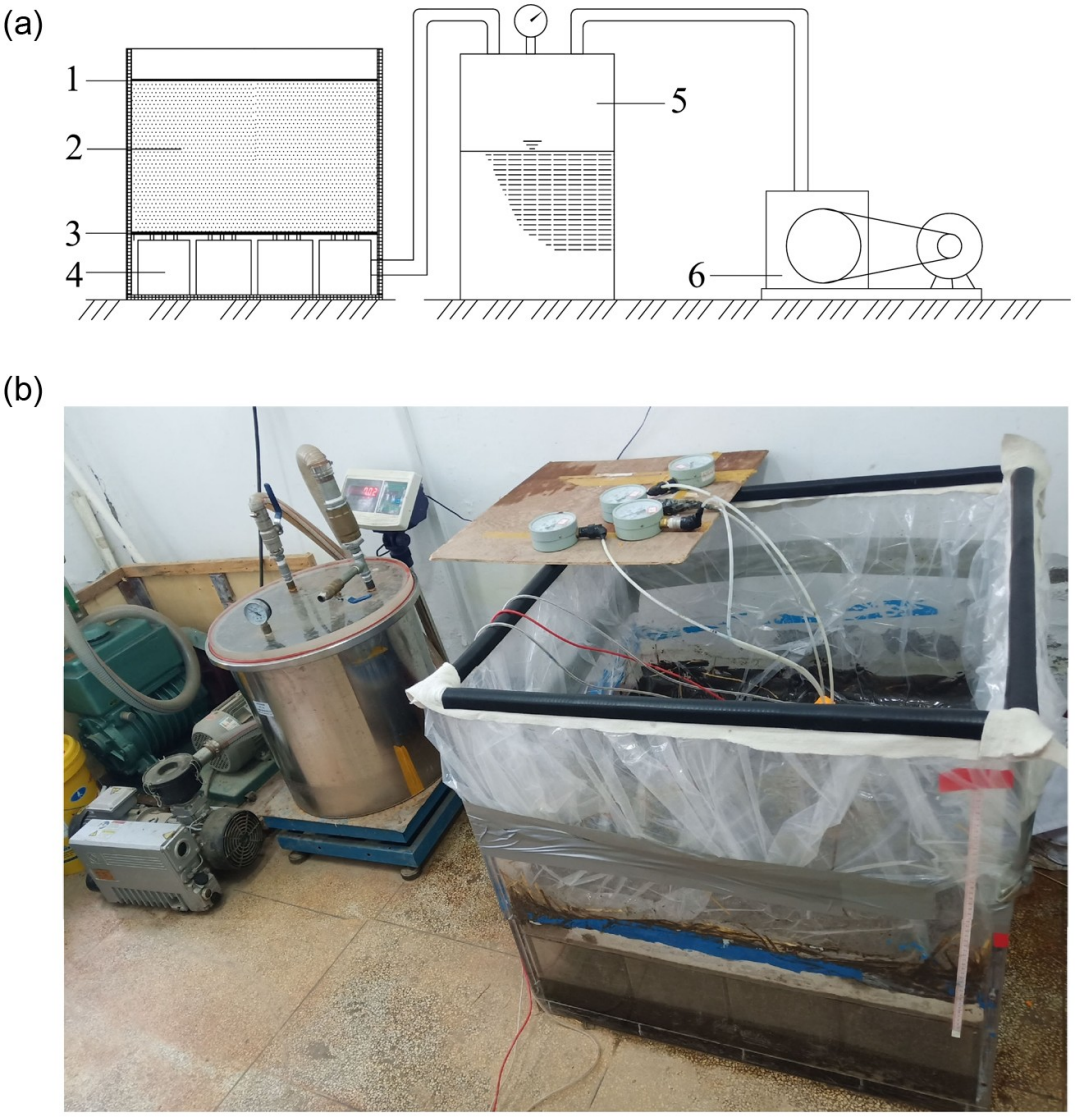
Sludge dewatering test device for single-drainage conditions. (a) Schematic diagram of the test model; (b) picture of the test model. 1- Sealing membrane; 2- sludge; 3- permeable geotextile; 4- vacuum negative pressure; 5- gas-water separator; 6- vacuum pump.

**Fig 4:**
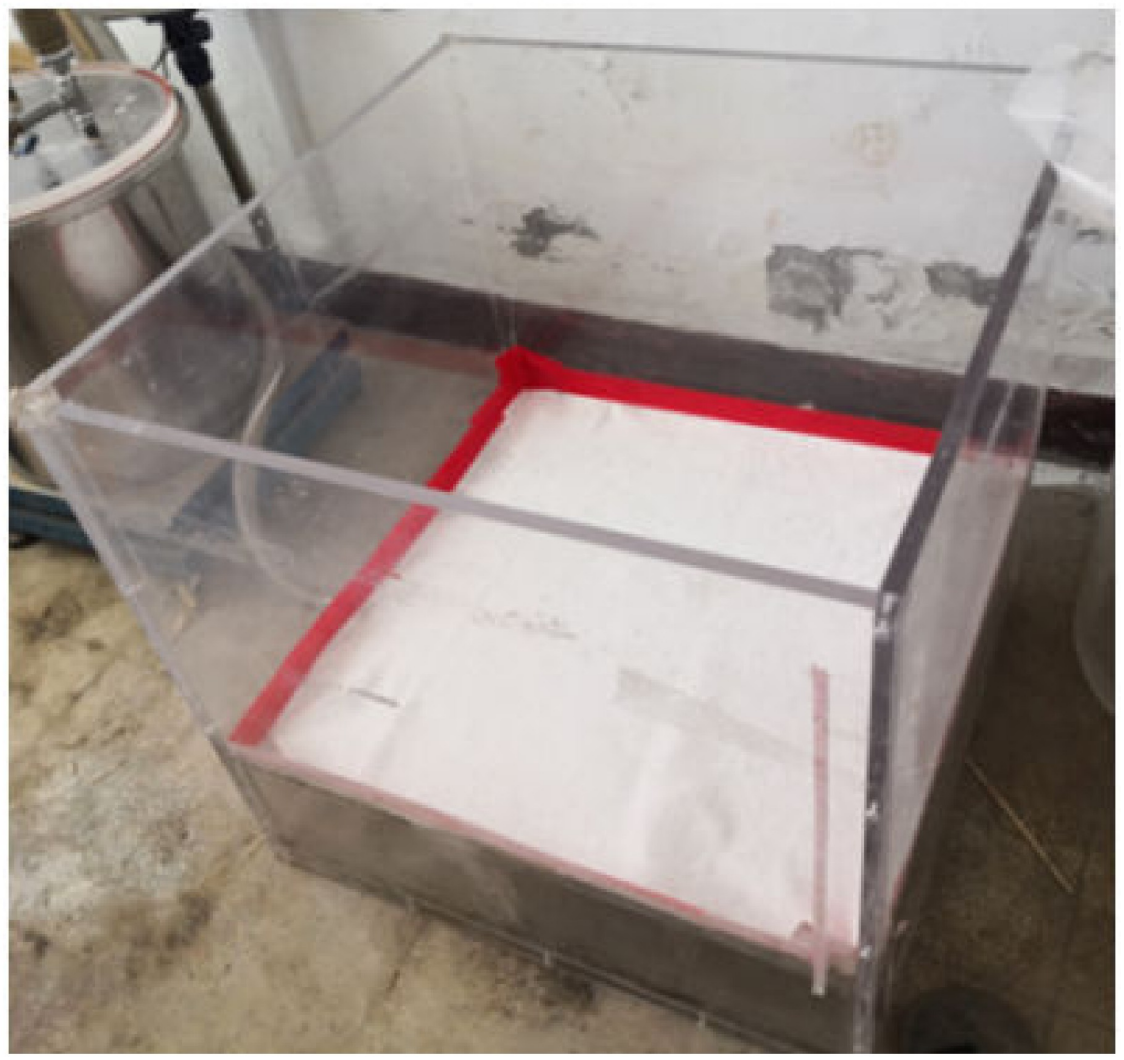
Layout of permeable geotextile layer.

The sludge dewatering test device for single-drainage conditions is shown in Fig 3. The model box consisted of a partition board, permeable geotextile, sludge layer, and sealing membrane from the bottom to top. A permeable geotextile layer was laid on the partition board as the filtration bed, and the edge of the geotextile was pasted and sealed with the inner side of the box wall, as shown in Fig 4. Then, the sludge was mixed evenly and discharged onto a permeable geotextile. The thickness of the sludge layer was 30 cm. The sludge layer’s upper surface was covered with a sealing membrane and sealed with the four walls of the model box.

**Fig 5:**
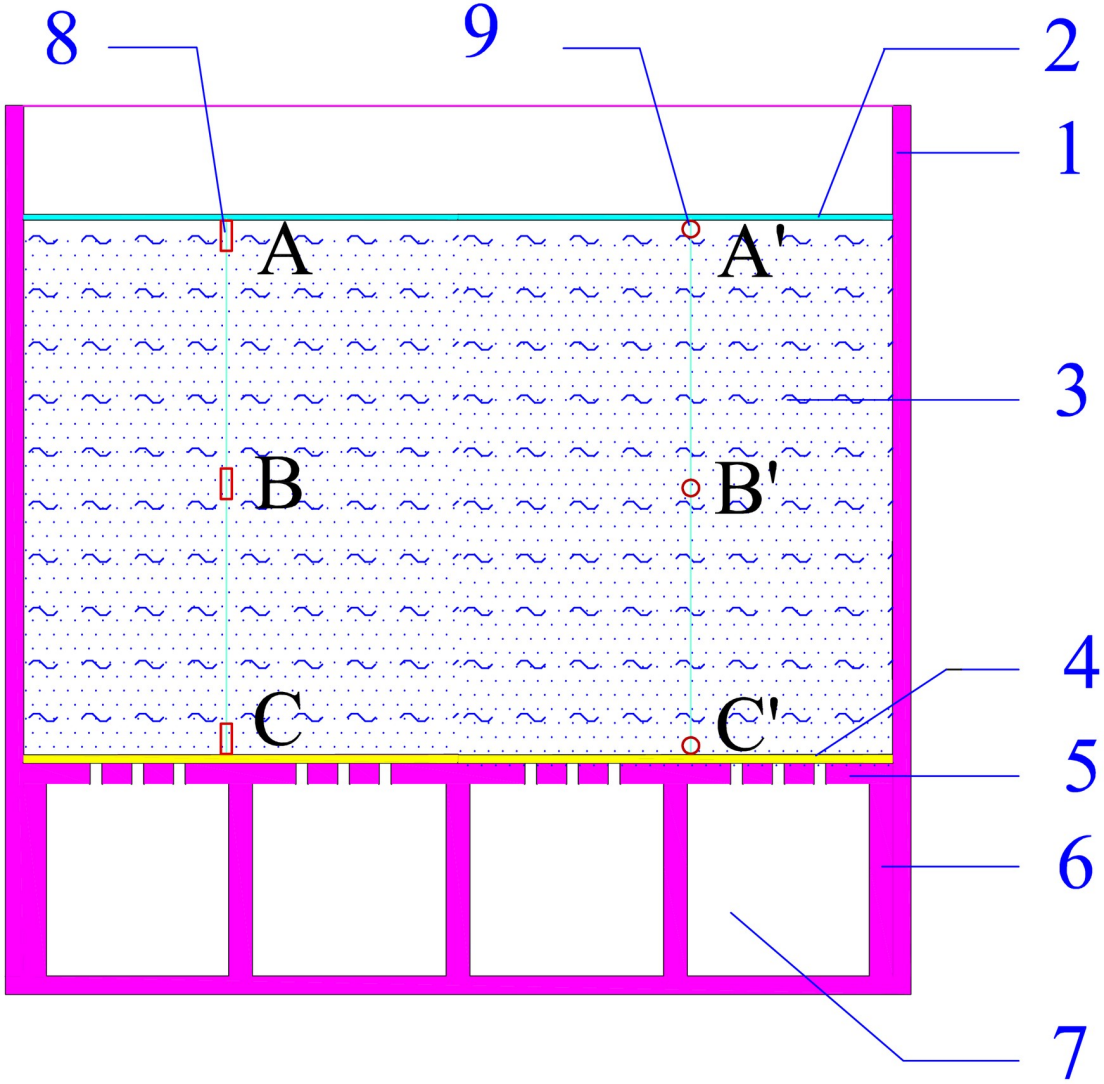
The locations of the sensors. 1-Model box; 2-sealing membrane; 3-sludge; 4- permeable geotextile; 5- partition board; 6- support; 7- hollow space; 8- pore pressure gauges; 9- vacuum probes.

The scale was set on the transparent model box’s outer wall to monitor the changes of the liquid level and the thickness of the sludge layer; the gas-water separator was placed on the precision electronic scale to measure the amount of sludge discharged. The vacuum probes and micropore water pressure gauges were embedded at the top, middle, and bottom of the sludge layer. The locations of the sensors are shown in Fig 5.

#### 1.2.2. Sludge dewatering for double-drainage conditions

**Fig 6:**
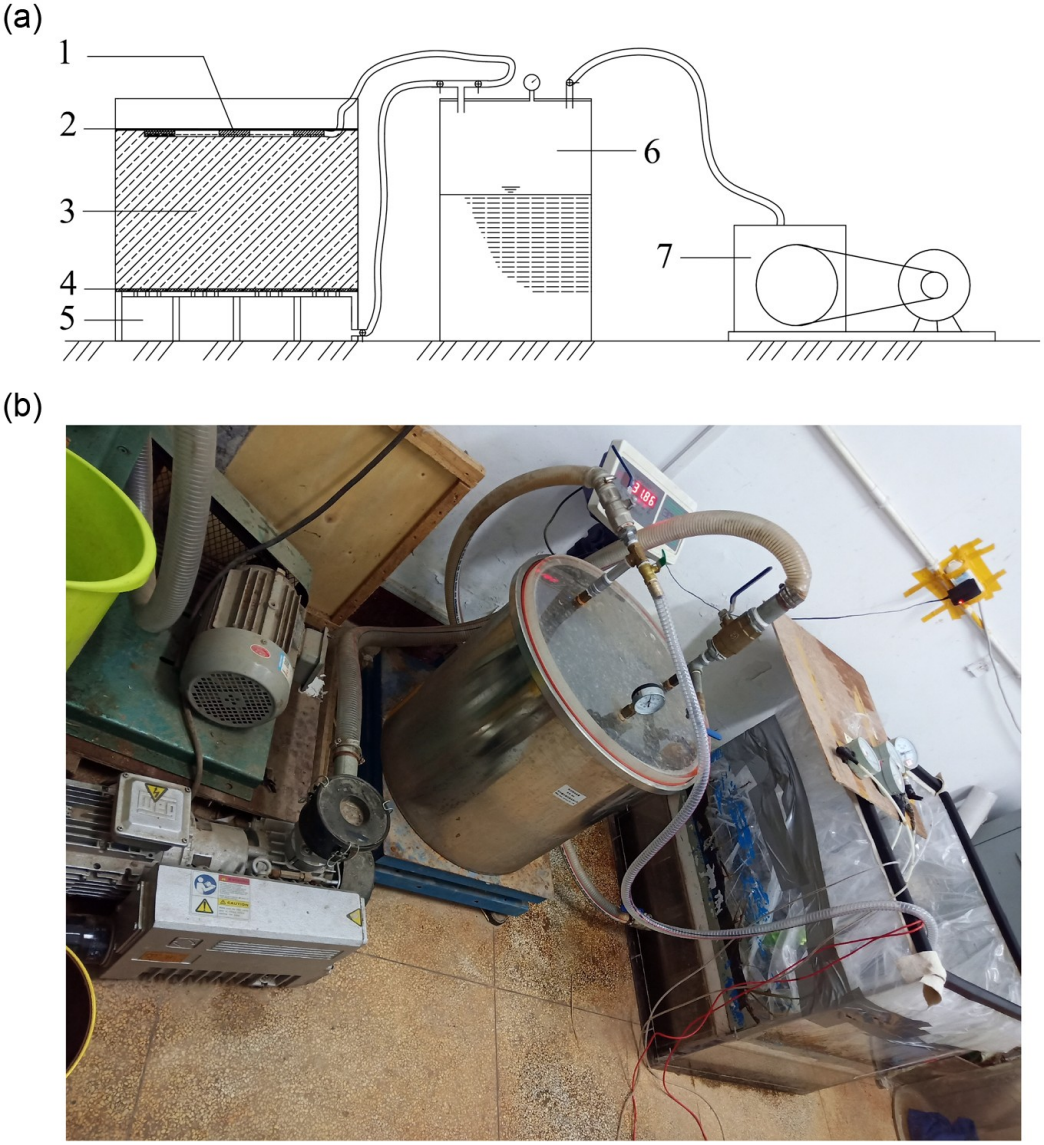
Sludge dewatering test device for double-drainage conditions. (a) Schematic diagram of the test model; (b) picture of the test model. 1- Drainage belts; 2- sealing membrane; 3- sludge; 4- permeable geotextile; 5- vacuum negative pressure; 6- gas-water separator; 7- vacuum pump. The layout of the drainage board is shown in Fig 7. The monitoring methods of the sludge level and sludge dewatering quantities were the same as those of sludge dewatering for single-drainage conditions. The locations of the sensors are shown in Fig 5.

The sludge dewatering test device for double-drainage conditions with a vacuum negative pressure load at the bottom is shown in Fig 6. The model box consisted of the partition board, permeable geotextile, sludge layer, plastic drain belt, and sealing membrane from bottom to top. A layer of permeable geotextile was laid on the partition board as the filtration bed, and the edge of the geotextile was pasted and sealed with the inner side of the box wall. Then, the sludge was mixed evenly and discharged onto a permeable geotextile. The thickness of the sludge layer was 30 cm. The drainage belts were placed on the sludge layer’s upper surface and covered with a sealing membrane.

**Fig 7:**
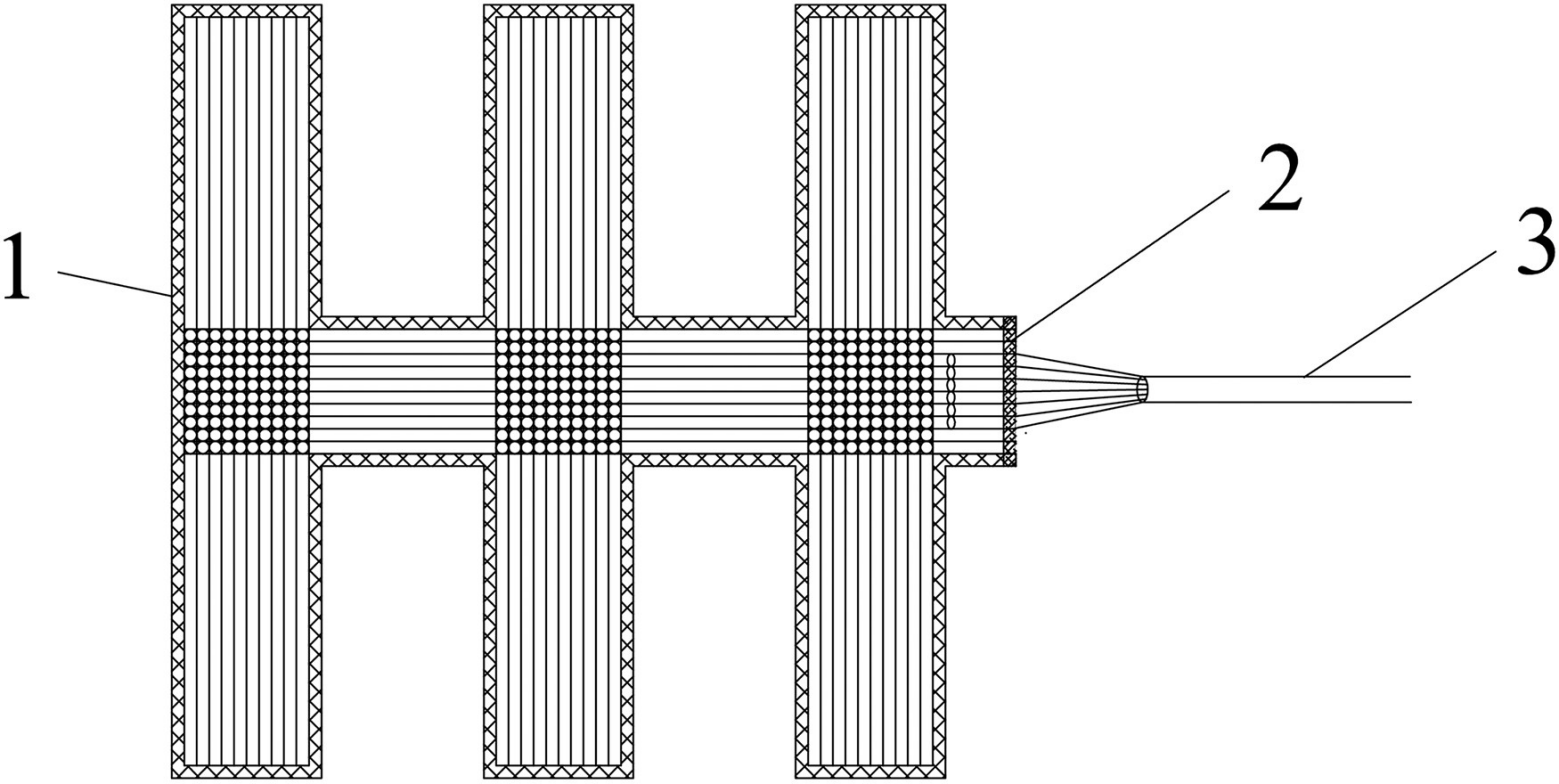
Layout of the drainage belts. 1- Drainage belts; 2- connection; 3- hose.

**Fig 8:**
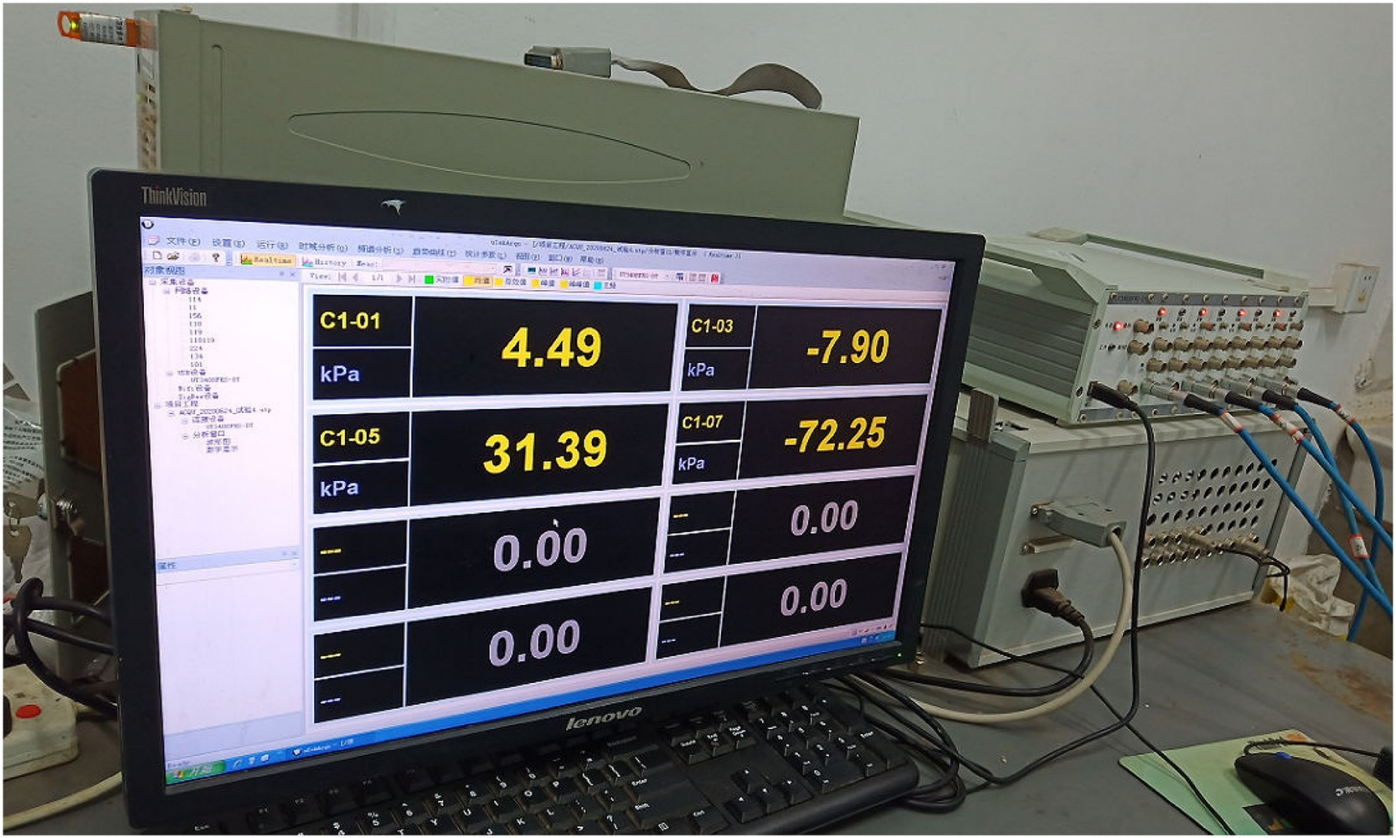
Test data collecting.

After the test preparation, the vacuum pump was started, and the test data were recorded, as shown in Fig 8. To ensure the comparability of the tests, the test time of the two groups was the same. Each of them was terminated in 24 hours, and sludge samples were taken to measure the final moisture content.

## 2. Results and discussion

### 2.1. The vacuum negative pressure load at the bottom

**Fig 9:**
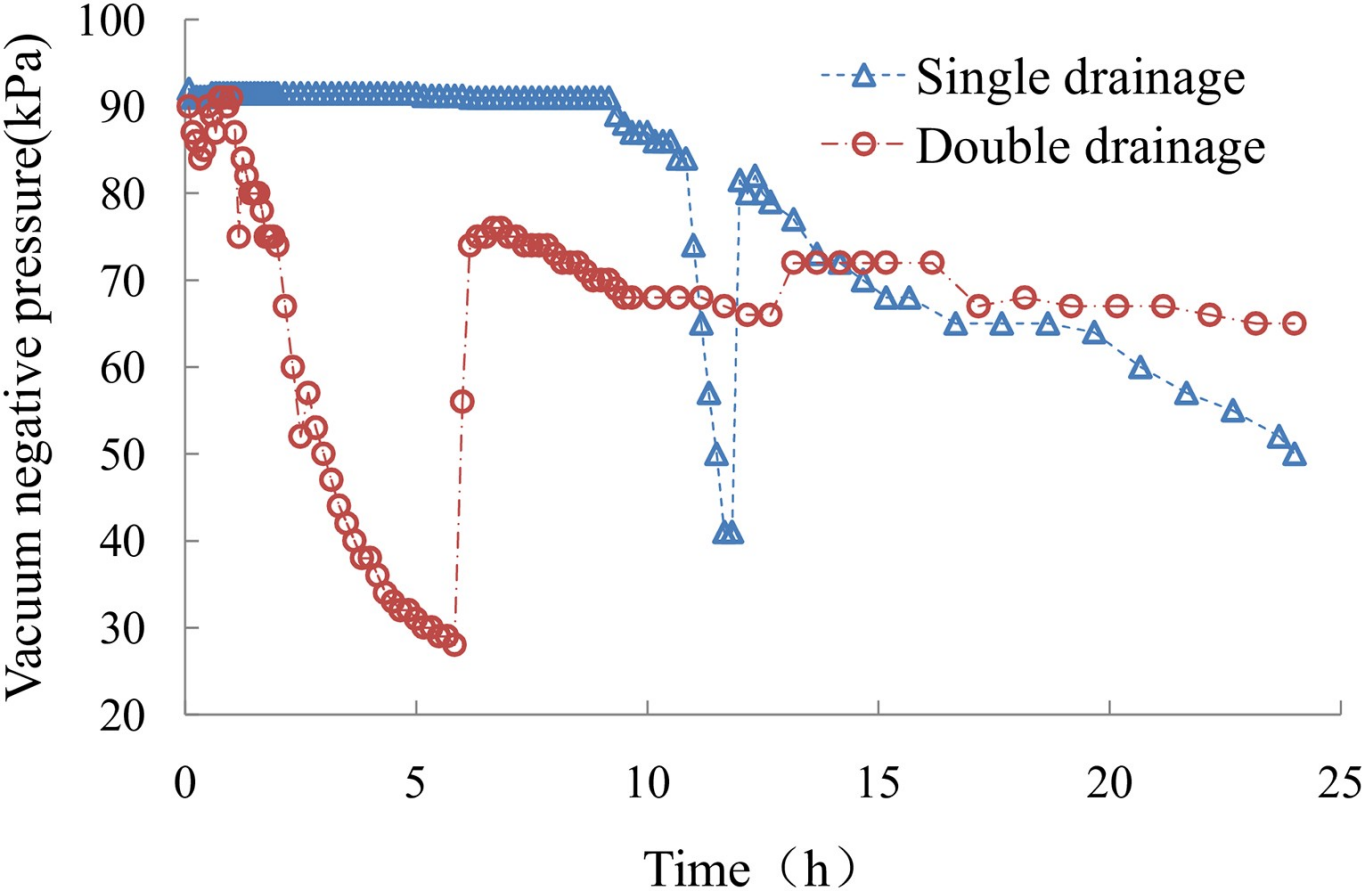
The vacuum negative pressure load at the bottom.

The curve of the vacuum negative pressure load at the bottom is shown in Fig 9. Fig 9 shows that the negative vacuum pressure at the bottom reached 91 kPa in the early stage of the sludge one-way drainage test. When the sludge was loaded for 11.5 hours, vacuum leakage occurred, and the bottom vacuum negative pressure decreased to 41 kPa. Then, the negative vacuum pressure returned to 80 kPa after remedial treatment. Due to poor sealing, the negative vacuum pressure gradually decreased to approximately 50 kPa. In the early stage of the double-drainage test, the negative vacuum pressure at the bottom was progressively reduced to 30 kPa due to the test equipment’s slow vacuum leakage. The vacuum pressure recovered to approximately 75 kPa when the equipment was rechecked and processed and was maintained at approximately 65 kPa until the end of the test.

### 2.2. Comparative analysis of sludge dewatering extent

**Fig 10:**
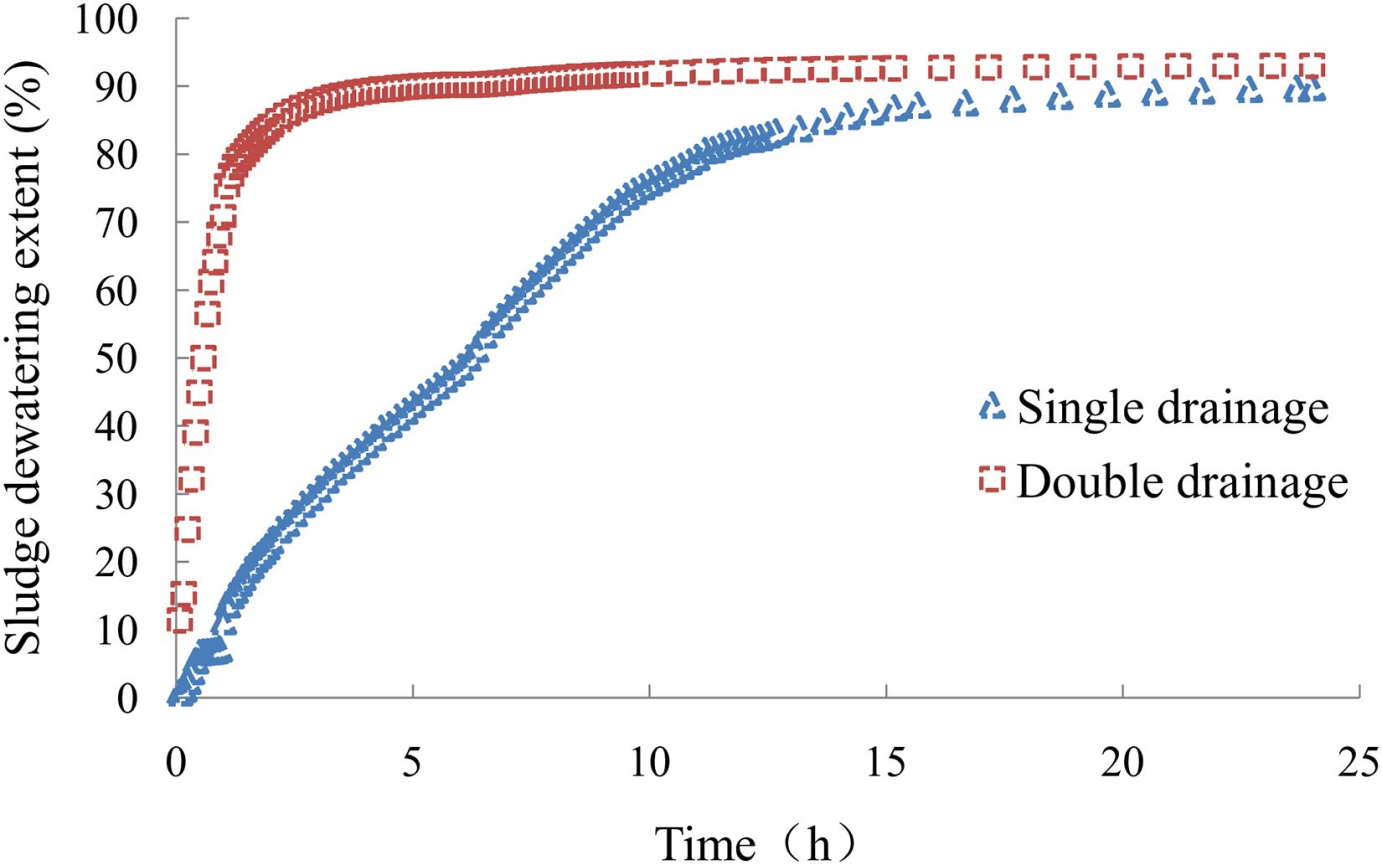
The curve of sludge dewatering extent.

The curve of sludge dewatering extent with a vacuum negative pressure load at the bottom is shown in Fig 10. The maximum sludge dewatering extent for single-drainage conditions was 89.8%, faster in the early test stage and slower in the late test stage. The maximum sludge dewatering extent for double-drainage conditions was 93.0%. Sludge dewatering under double-drainage conditions was very fast in the early test stage but very slow in the late test stage. Sludge dewatering under double-drainage conditions was much better than that for single-drainage conditions in the early test stage. The sludge dewatering extent under double-drainage conditions reached 90.0% in 5 hours, which exceeded the final effect observed under single-drainage conditions. The dewatering efficiency for double-drainage conditions was far higher than that under single-drainage conditions with a vacuum negative pressure load at the bottom.

### 2.3. Comparative analysis of sludge moisture content

**Fig 11:**
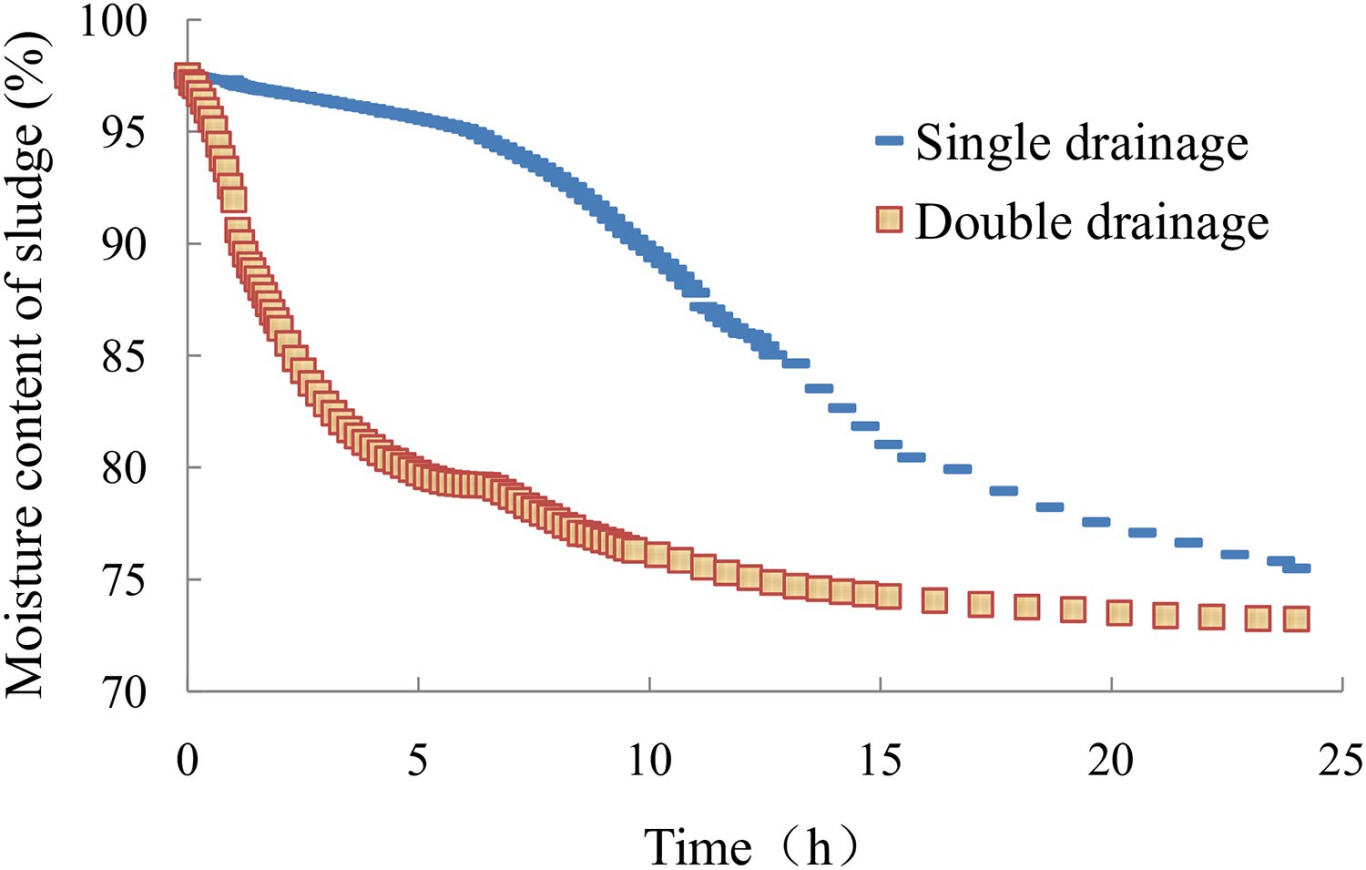
Variation in sludge moisture content.

The sludge moisture content curve with a vacuum negative pressure load at the bottom is shown in Fig 11. The initial moisture content of the sludge was 97.5% before the test. The final moisture content under single-drainage conditions decreased to 75.5% at the end of the test. The sludge moisture content under single-drainage conditions slowly decreased in the early and late test stages and rapidly decreased in the middle test stage. The final sludge moisture content under double-drainage conditions decreased to 73.2%. The sludge moisture content under double-drainage conditions rapidly decreased in the early test stage and then decreased slowly. Fig 11 shows that the sludge moisture content under double-drainage conditions decreased much faster than under single-drainage conditions in the early test stage, and the dewatering efficiency was much higher. The results show a significant difference between the sludge moisture content curve under double-drainage conditions and that for under single-drainage conditions. The sludge moisture content curve for single-drainage conditions presented an "S" shape, and there was an inflection point in the moisture content curve. However, the sludge moisture content curve under double-drainage conditions was similar to a logarithmic curve, and there was no inflection point.

### 2.4. Comparative analysis of sludge dewatering velocity

**Fig 12:**
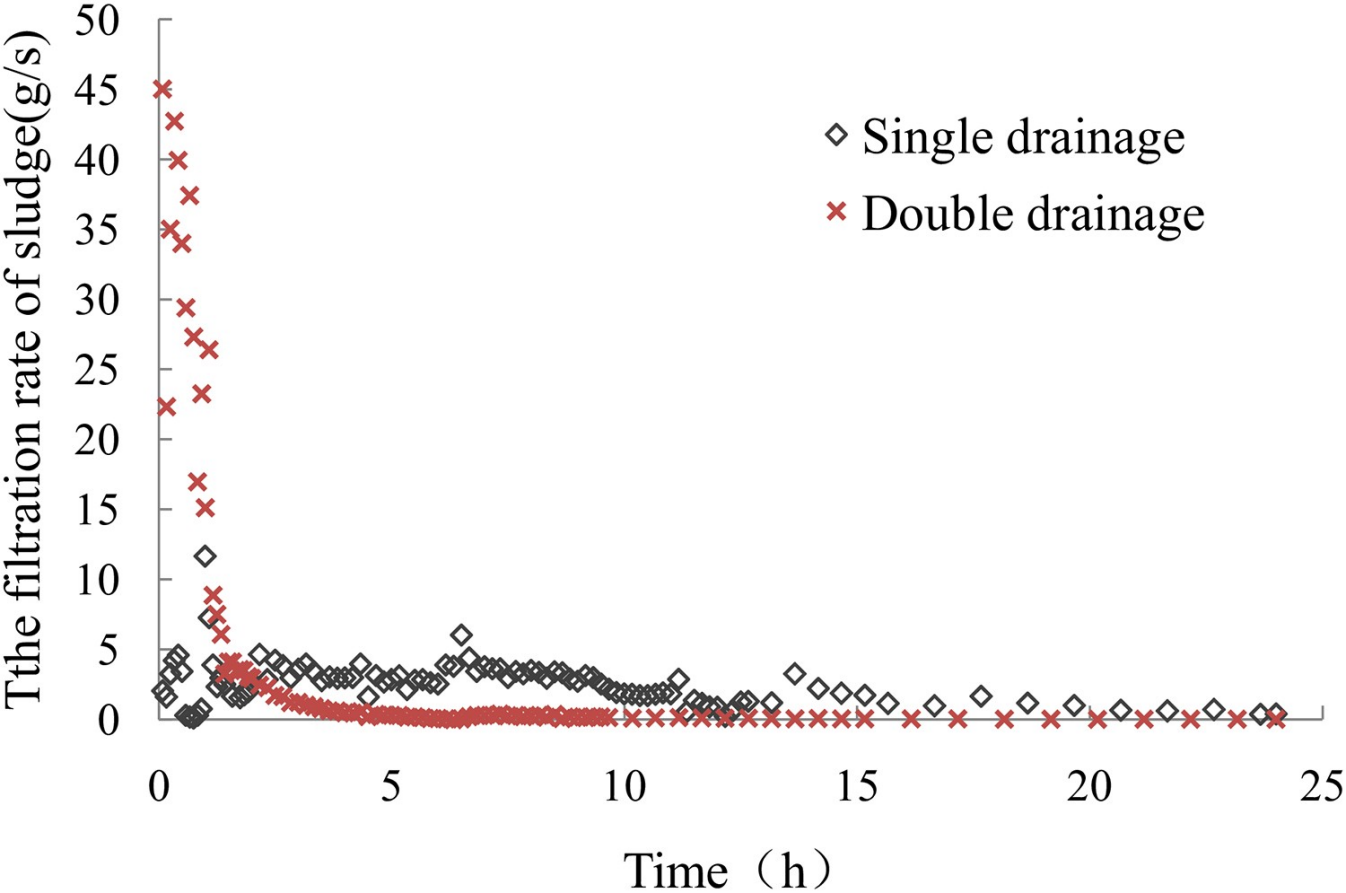
The velocity of sludge dewatering.

The curve of the sludge dewatering velocity with a vacuum negative pressure load at the bottom is shown in Fig 12. The sludge dewatering velocity for single-drainage conditions was significant at the beginning of loading and decreased rapidly in the early test stage, fluctuated in a certain range, and finally decreased gradually in the late test stage. The sludge dewatering velocity under double-drainage conditions was very high at the beginning of loading, far greater than that for single-drainage conditions. The sludge dewatering velocity under double-drainage conditions was lower than that under single-drainage conditions after 2 hours of loading. It can be seen from the sludge dewatering velocity curve that the sludge dewatering efficiency was higher with double drainage than with single drainage.

### 2.5. Comparative analysis of the vacuum degree in the sludge layer

**Fig 13:**
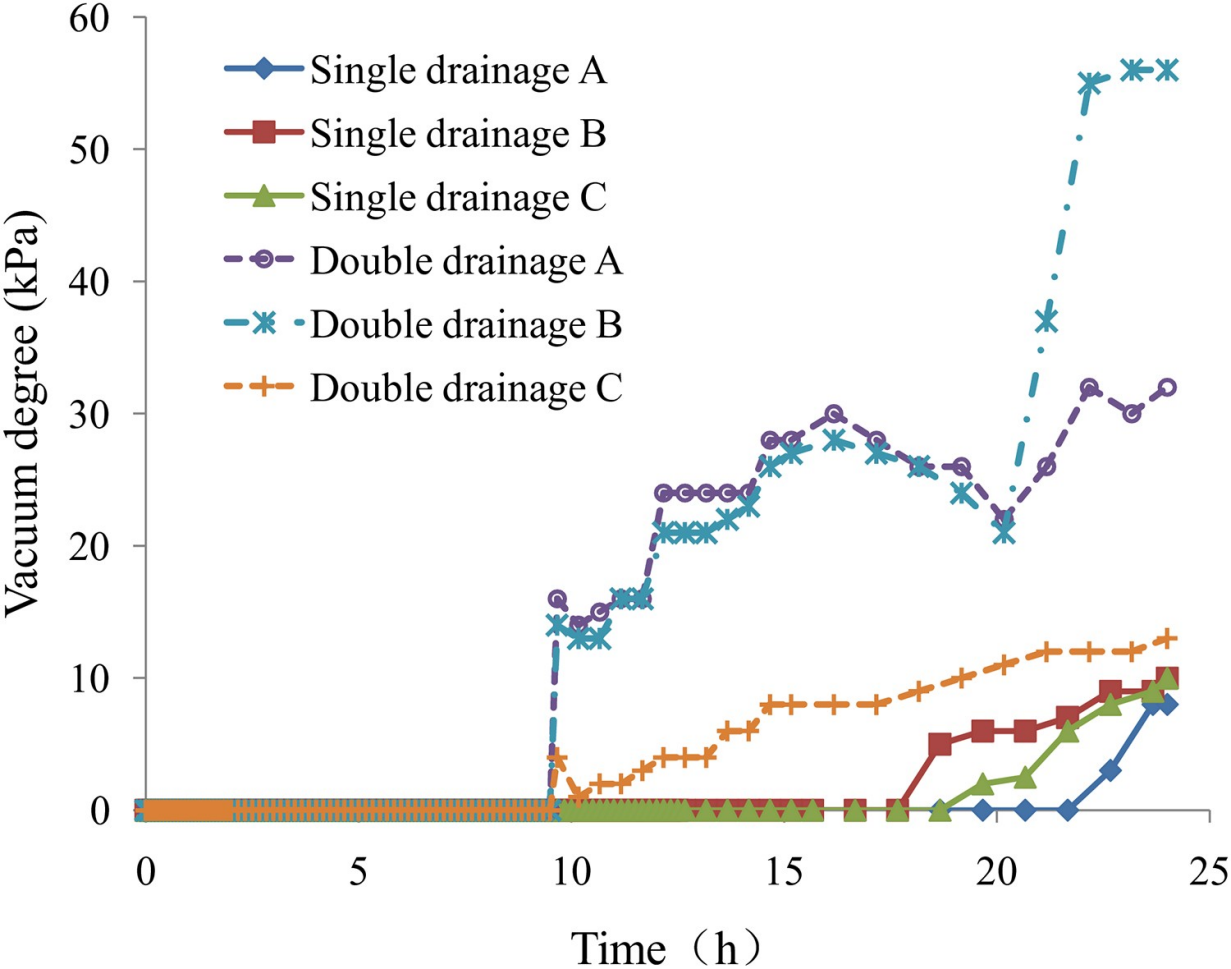
Vacuum degree in the sludge layer.

In the tests, three groups of vacuum probes were set at the top, middle, and bottom of the sludge layer (see Fig 5 for details). The curves of the vacuum degree of sludge dehydration are shown in Fig 13. The vacuum in the sludge under single-drainage conditions occurred in 18 ~ 22 hours, and the vacuum degree was below 10 kPa. The vacuum in the sludge under double-drainage conditions occurred in 9 ~ 10 hours, and the vacuum degree was much higher than that under single-drainage conditions. The vacuum attenuation under single-drainage conditions was more severe than that under double-drainage conditions.

The phenomenon above shows that the pore water inside the sludge has the effect of "water sealing", which prevents the vacuum from spreading in the sludge. When the sludge supernatant disappeared, the vacuum began to displace the pore water in the sludge pores, and a vacuum was formed. Compared with that in single sludge drainage, the vacuum in the sludge under double-drainage conditions formed earlier, the vacuum degree was larger, and the sludge dewatering effect was better.

### 2.6. Comparative analysis of pore water pressure

**Fig 14:**
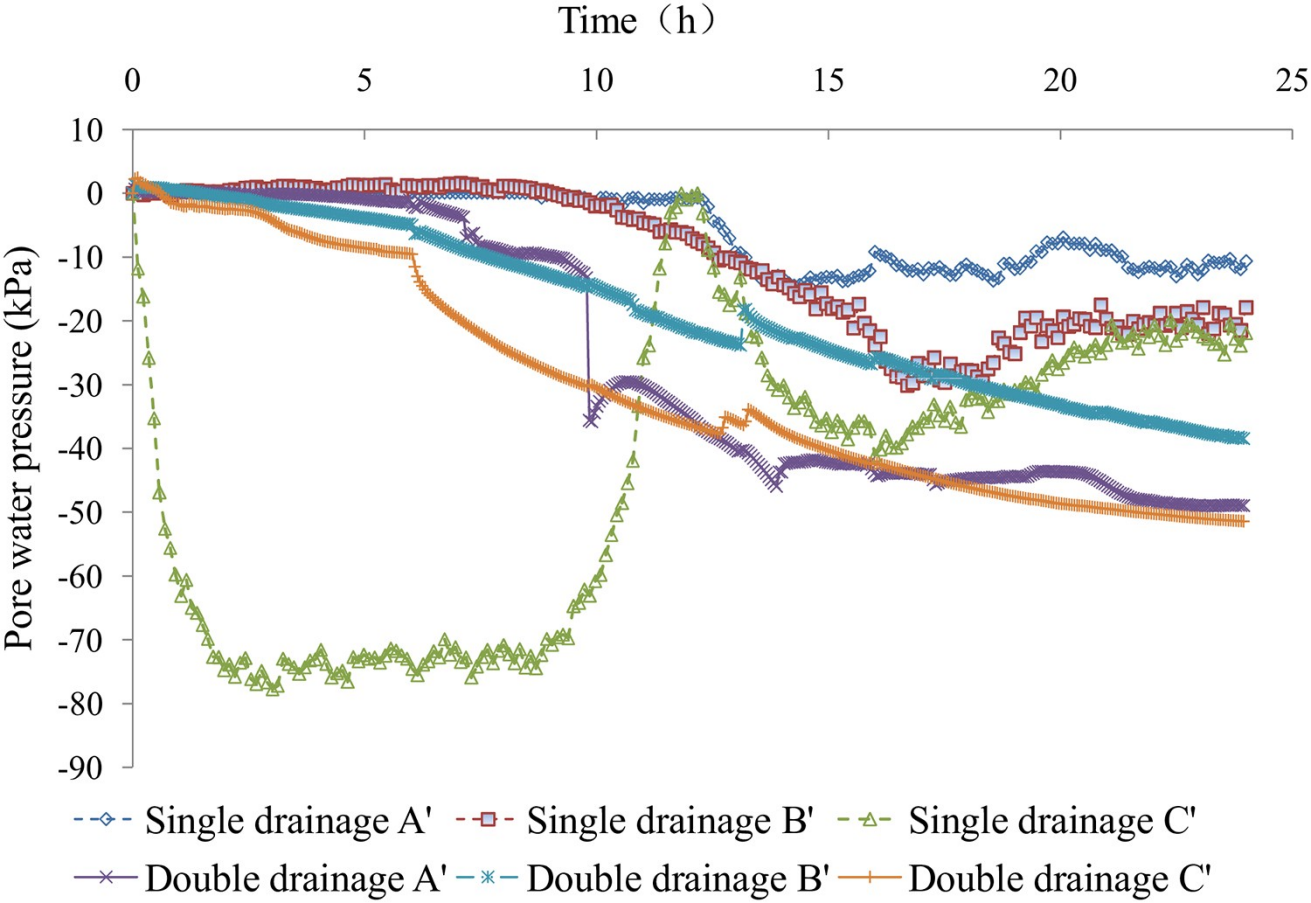
Pore water pressure in the sludge.

In the tests, three groups of pore water pressure gauges were set at the top, middle, and bottom of the sludge layer (see Fig 5 for details), consistent with the vacuum degree probe positions. The pore water pressure curves are shown in Fig 14.

The absolute value of the negative pore water pressure in the sludge for single-drainage conditions was very large in the bottom of the sludge layer and small in the top of the sludge layer, which indicates that the negative pressure transferred gradually from the bottom of the sludge layer to the top. The negative pore water pressure in the middle and top of the sludge layer under single-drainage conditions decreased slowly in the early and late test stages and rapidly in the middle test stage. There is an inflection point in the pore water pressure curve. Because the bottom of the sludge layer was close to the surface of the vacuum negative pressure load and there was little clogging of the permeable geotextile by sludge in the early test stage, the pore water pressure decreased rapidly, as shown in Fig 14. The negative pressure increased quickly in 11.5 hours of loading due to the test equipment’s vacuum leakage. Thereafter, the pore water pressure decreased gradually.

The pore water pressure at the top of the sludge layer decreased rapidly after 10 hours of loading, and the value was approximately equal to the negative vacuum pressure at the bottom of the sludge layer. The time at which the pore water pressure suddenly decreased was consistent with the occurrence of a vacuum. The negative forces were then transferred from the top and bottom of the sludge layer to the middle of the sludge layer at the same time.

The pore water pressure under double-drainage conditions was less than that under double-drainage conditions at the same position (except for the special circumstances of early pore water pressure at the bottom of single-drainage sludge dewatering). Compared with that under single-drainage sludge conditions, the excess pore water pressure under double-drainage conditions dissipated faster, the sludge dewatering efficiency was higher, and the dewatering effect was better.

## 3. Conclusions

In this study, sludge dewatering tests under single- and double-drainage conditions with a vacuum negative pressure load at the bottom were studied, and the test results were compared and analyzed. The conclusions are as follows.

1. The sludge moisture content under single- and double-drainage conditions with a vacuum negative pressure load at the bottom decreased from the initial 97.5% to 75.4% and 73.2%, respectively, without sludge conditioning. The sludge changed from a slurry to a dense cake after dewatering, and the effect of sludge dewatering was good. The results showed that the sludge moisture content for single-drainage conditions decreased slowly in the early and late test stages but decreased rapidly in the middle test stage. The sludge moisture content curve under single-drainage conditions showed an "S" shape, and there was an inflection point. The sludge moisture content curve under double-drainage conditions was similar to the logarithmic curve without an inflection point.
2. The sludge dewatering velocity under single-drainage conditions was high at the beginning of loading, decreased rapidly in the early test stage, fluctuated, and finally decreased gradually in the late test stage. The sludge dewatering velocity under double-drainage conditions was far greater than that for single-drainage conditions and decreased very fast in the early test stage. At the same time, the sludge dewatering extent under double-drainage conditions was much greater than under single-drainage conditions in the early test stage. Therefore, the efficiency of sludge dewatering under double-drainage conditions was much higher than that under single-drainage conditions.
3. Vacuum conditions did not form at the beginning of loading, which indicated that the pore water inside the sludge had the effect of "water sealing" to prevent the spread of vacuum conditions in the sludge. The vacuum in the sludge under double-drainage conditions appeared earlier than under single-drainage conditions, and the vacuum degree was larger. The vacuum attenuation of sludge dewatering was more severe with single drainage than with double drainage.
4. The excess pore water pressure dissipated slowly in the early and late test stages and rapidly in the middle test stage. The inflection point of the pore water pressure curve appeared in the middle test stage. The pore water pressure under single-drainage conditions in the top and middle in the sludge layer increased at first and then decreased in the early stage of loading, resembling the Mandel effect. Compared with that of single sludge drainage, the excess pore water pressure under double-drainage conditions dissipated faster, the sludge dewatering efficiency was higher, and the dewatering effect was better.
